# Economic Uses, Specific Metabolites and Molecular Biology Research of the Genus *Zanthoxylum*

**DOI:** 10.3390/foods15030540

**Published:** 2026-02-03

**Authors:** Xing-Dou Wang, Wei He, Wen-Jun Wang, Yuan-Yuan Ren, Nian Wang, Zhi-Hua Hou, Na Guo, Xiao-Qiao Zhai, Guo-Qiang Fan

**Affiliations:** 1 Institute of Paulownia, College of Forestry, Henan Agricultural University, Zhengzhou 450002, China; 18703489369@163.com; 2 Forest Tree Germplasm Resources Innovation and Utilization Key Laboratory of Henan, Forestry Academy of Henan, Zhengzhou 450002, Chinauser7117@163.com (X.-Q.Z.)

**Keywords:** *Zanthoxylum*, economic uses, specific metabolites, flavor, biosynthetic pathway

## Abstract

*Zanthoxylum* plants are a perennial economic crop which have garnered significant attention owing to their distinctive smell and taste. Their main flavor characteristics include a numbing sensation, bitterness, and aroma, which are mostly contributed by secondary metabolites, including alkaloids, flavonoids, and terpenes. As an important spice and a natural food additive, *Zanthoxylum* has broad application prospects and economic value in the production of food, medicine, animal feed, and raw chemical materials. This review aimed to provide a comprehensive overview of the economic uses and main flavor metabolites of *Zanthoxylum*. Furthermore, molecular biology research into the plant was summarized in detail. This will provide a reference for the future development and utilization of *Zanthoxylum*, and reveal the molecular mechanisms involving the biosynthesis of its flavor metabolites.

## 1. Introduction

*Zanthoxylum* is a genus within the Rutaceae family, which contains over 200 species. It is a shrub/tree with a long history of cultivation [1]. It is native to warm temperate and subtropical regions. In Asia, this genus is frequently found in the Himalayan region, as well as Central Asia, South Asia, Southeast Asia, and East Asia. Depending on the pigment of the peel, *Zanthoxylum* is divided into two categories: green pepper (*Z. schinifolium* Siebold & Zucc, *Z. armatum* DC., and others) and red pepper (such as *Z. bungeanum* Maxim.) [2,3]. *Zanthoxylum* is one of the most important spices and medicinal materials, and it is enjoyed by people worldwide [4]. The sprout, seed, and peel of *Zanthoxylum* are edible [5,6,7], with oil extract obtained from seeds, while the bark has medicinal purposes.

Flavor is the sense of smell and taste formed by chemicals that stimulate the central nervous system. It is one of the most impactful sensory impressions of products. To meet the increasing demand of consumers for products, flavor has been placed in an important position by the Flavor and Extract Manufacturers Association (FEMA) [8]. In *Zanthoxylum*, the flavors are mainly divided into three types: numbness, bitterness, and aroma. These flavors are caused by secondary metabolites, such as alkaloids, flavonoids, and terpenes. Alkaloids are mainly responsible for numbness, coumarins, amino acids, and flavonoids are the main contributors of bitterness, and volatile oils rich in terpenes are largely responsible for aroma [9].

Different *Zanthoxylum* species have their own specific primary and secondary metabolites. In plants, primary metabolites are essential for growth and include sugars, organic acids, amino acids, and proteins, while secondary metabolites relate to plant interactions with the surrounding environment and mainly include phenylpropanoids, terpenoids, and alkaloids [10]. Metabolites are often seen as a bridge between genotypes and phenotypes, and changes in the levels of metabolites can directly reveal functional genes; thus, it is more effective to elucidate related biochemical and molecular mechanisms [11]. *Zanthoxylum* has evolved multiple metabolites [12,13] that provide nutritional benefits in foods, and these substances are also of interest in agriculture, medicine, and other fields.

With the development of molecular biology, researchers have made progress in the molecular biological research of *Zanthoxylum* [14,15]. Studies related to genomics, transcriptomics and metabolomics have been conducted on *Zanthoxylum* for a deep analysis of its potential value in molecular biology, especially in biological synthesis. However, up to now, little research has been systematically conducted in this field. In this review, the economic uses, specific metabolites and biosynthetic pathways of the main flavor metabolites in *Zanthoxylum* were summarized. The developmental directions of plants in the *Zanthoxylum* genus in the fields of food, industry, and medicine were also explored, aiming to provide a reference for the development and utilization of important economic crops.

## 2. Economic Uses

### 2.1. Foods

The use of *Zanthoxylum* as a spice spans over 2000 years in Chinese history and it plays a significant role in the culinary traditions of Southeast Asia. In Chinese cuisine, the peel of *Zanthoxylum* is a crucial condiment that adds flavor, color, and other sensory elements. It is an essential auxiliary seasoning in almost all Chinese recipes and a key ingredient in hot pot bases, which provides a numbing and aromatic flavor foundation for pot foods [16,17,18]. *Zanthoxylum* is commonly used as a seasoning in dumplings, and the young shoots can be utilized as ingredients [19]. Furthermore, *Zanthoxylum* extract can be combined with chitosan to create a polymer shell, which enhances the shelf life of fruits [20]. Consumer acceptance of foods relies on their flavor, and *Zanthoxylum* also contains aromatic and valuable terpenoids, such as limonene, myrcene, and linalool, which are crucial raw materials in various industries [21,22].

### 2.2. Medicines

*Zanthoxylum* has been utilized for centuries in traditional Chinese medicine for its therapeutic properties. According to the Chinese Pharmacopoeia, *Zanthoxylum* peels can be used as a Chinese herbal medicine. The conventional oral dosage is 3 to 6 g, with variable doses given, such as Ebony Pill, Tong Luo Qu Tong Gao, and Ke Tong Ding [23]. *Zanthoxylum* has demonstrated various pharmacological effects, including antibacterial [24], anti-inflammatory [25,26], analgesic [27], antiviral [28], and anti-tumor effects [29]; anti-platelet aggregation [30]; antioxidant activities [31]; and hypolipidemic activity [32]. *Z. bungeanum* can inhibit bacterial growth by disrupting the permeability and integrity of bacterial cell membranes [24]. Extracts of *Z. austrosinense* exhibit anti-proliferative effects on human tumor cells, with IC50 values ranging from 0.85 ± 0.06 to 29.56 ± 0.17 µM [25]. *Z. bungeanum* extract has the highest antioxidant activity (75.57%) compared with extracts from five species of the Rutaceae family [31]. Moreover, *Zanthoxylum* is also employed in the treatment of ailments in ethnic medicine across East Asia, the Americas, and Europe [33]. Additionally, *Zanthoxylum* extract is commonly utilized as both an insect repellent and herbicide. It possesses neurotoxic properties that can impact the development of mosquito larvae [34].

### 2.3. Animal Feed

*Zanthoxylum* seeds are often considered by-products, although they account for 60–70% of its total production. The seeds are rich in lipids, amino acids, and proteins, which makes them suitable as feed components. The incorporation of *Z. bungeanum* seeds into feed can enhance the rates of egg production and improve the quality of eggs in laying hens [35]. Furthermore, adding 5% *Z. bungeanum* seeds to feed does not negatively impact the growth of Jian carp [36].

### 2.4. Industrial Applications

**Figure 1:**
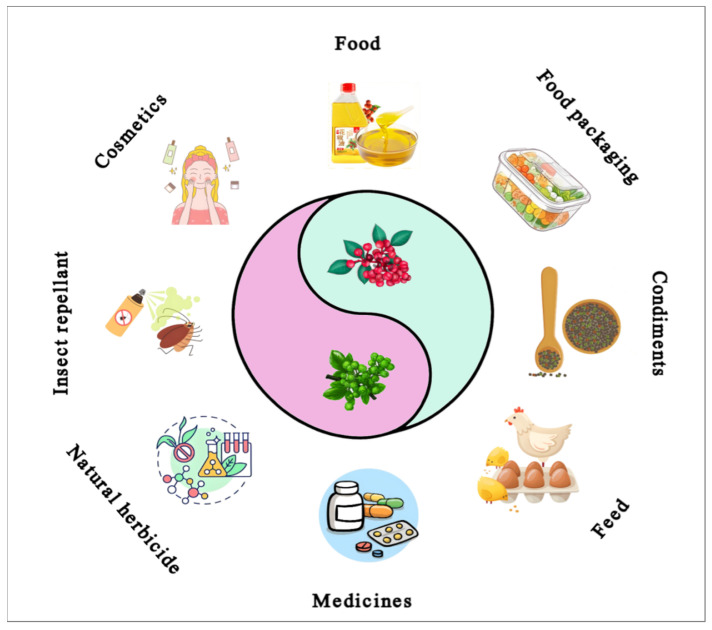
Economic uses and industrial applications of *Zanthoxylum*.

Advancements in methods to extract, separate, and identify metabolites have become more precise and wide-ranging. As a result, the applications of *Zanthoxylum* in various aspects of people’s lives are diversified, and the outstanding benefits of this plant have contributed to the development of numerous industries. Patent applications related to *Zanthoxylum* encompass areas such as food, medicine, cosmetics, agricultural production, feed processing, and mechanized manufacturing (Figure 1 and Table 1). The findings of these studies have significant implications for enhancing the value of *Zanthoxylum* applications, promoting its development, and improving human well-being.

## 3. Specific Metabolites

### 3.1. Terpenoids

**Figure 2:**
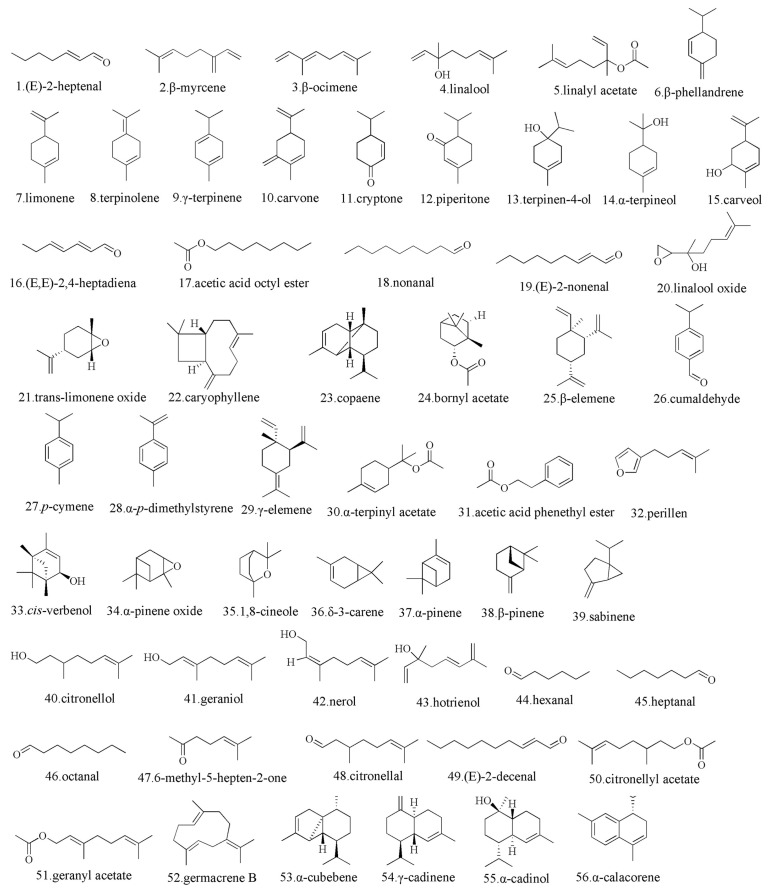
Chemical structures of terpenoids isolated from *Zanthoxylum* [38,40,41,42].

*Zanthoxylum* is abundant in aromatic oils [37], with the main aromatic compounds of linalool, limonene, geraniol, laurene, and cedrene [38,39]. These compounds are significantly higher in *Z. bungeanum* than *Z. armatum* [40]. During the maturation process, the content of monoterpenes and linalyl acetate significantly increases shortly after pollination and early fruit development, and gradually decreases as the fruit matures. The content of aldehydes also gradually decreases with fruit maturity [41]. The main terpenoids isolated from *Zanthoxylum* are shown in Figure 2. The terpenoids with the highest content in *Z. bungeanum* are limonene and linalool [40], which are volatile aromatic compounds rich in biological activity. Limonene scavenges free radicals, inhibits membrane lipid peroxidation, and exhibits anti-inflammatory effects [41]; for instance, treatment with 0.1 g/kg for 14 days had the strongest anti-inflammatory effect in mice ear models [42]. Furthermore, linalool can exert anti-tumor activity by inducing apoptosis, which leads to the generation of reactive oxygen species in liver cancer cells [43].

### 3.2. Alkaloids

**Figure 3:**
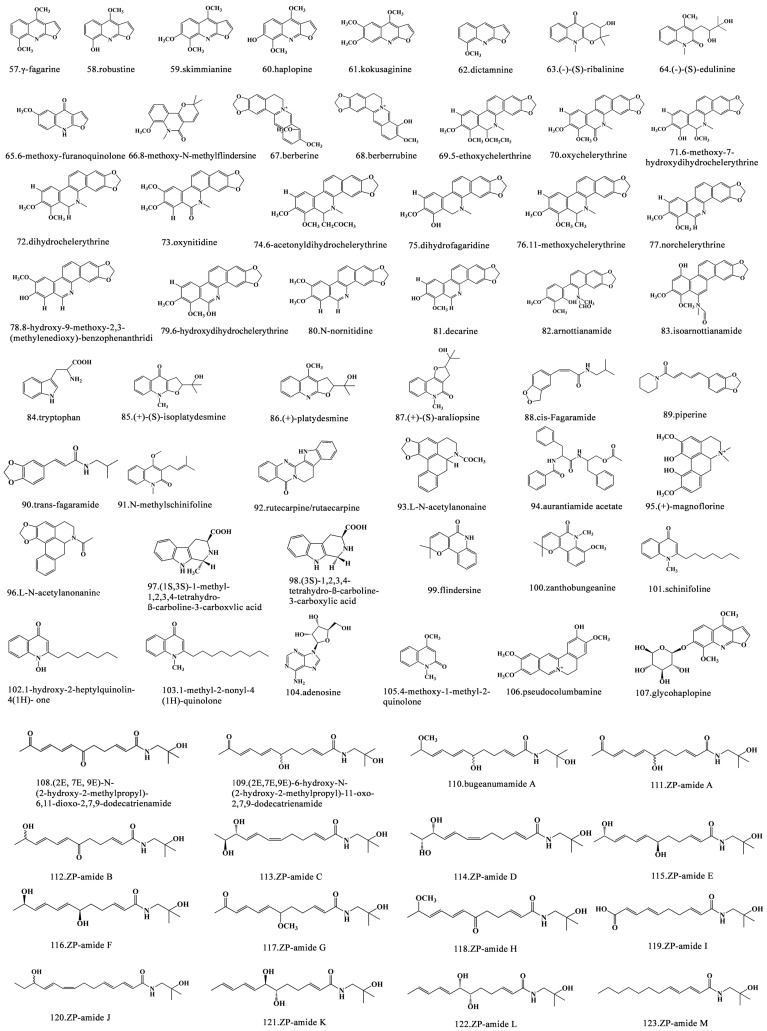

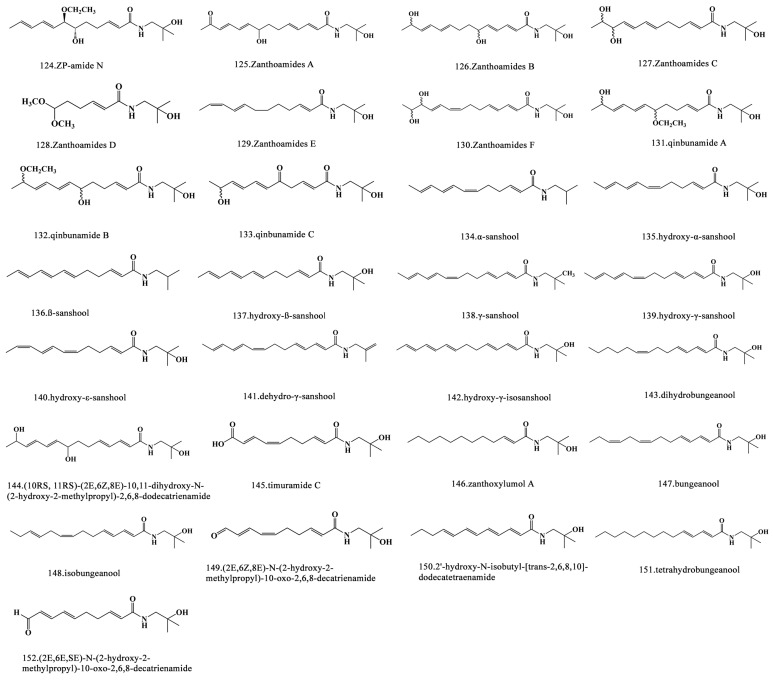
Chemical structures of alkaloid components isolated from *Zanthoxylum* [18,22,25].

Alkaloids are endogenous metabolites of sessile plants, and the 16,000 alkaloids identified to date constitute a highly diverse group of secondary metabolites [44]. In *Zanthoxylum*, over 200 alkaloids have been identified [45]. According to their chemical structures, *Zanthoxylum* alkaloids are mainly divided into four categories: (1) quinoline, (2) isoquinoline, (3) benzo[c]phenanthridine, and (4) quinolone [46]. The main alkaloids isolated from *Zanthoxylum* are shown in Figure 3. The numbing component of *Zanthoxylum* is fagaramide, which is a phenylpropylamino alkaloid commonly known as “sanshool” [47]. Sanshool was first identified by Murayama in 1913 [47] and its different configurations were discovered in 1982 [48]. Recently, dozens of sanshools have been isolated and identified from the peels of *Z*. *bungeanum* and *Z*. *schinifolium*, including hydroxy-α-sanshool, hydroxy-β-sanshool, hydroxy-ε-sanshool, hydroxy-γ-sanshool, δ-zanicosamine, and their alkaloid derivatives [49]. Hydroxy-α-sanshool in *Z. piperitum* can induce currents and Ca^2+^ influx in a capsazepine-dependent manner, which results in numbness in humans [50]. N-[2-(3,4-dimethoxyphenyl)ethyl]-3-phenyl-acrylamide alkaloid (WGX-50/GX-50) in *Zanthoxylum* is a well-known pepper extract, which can reduce wrinkles, relieve pain, and induce apoptosis of hepatoma cells in vitro [51,52,53]. Furthermore, it is an important drug candidate for the treatment of Alzheimer’s disease [54].

### 3.3. Flavonoids

**Figure 4:**
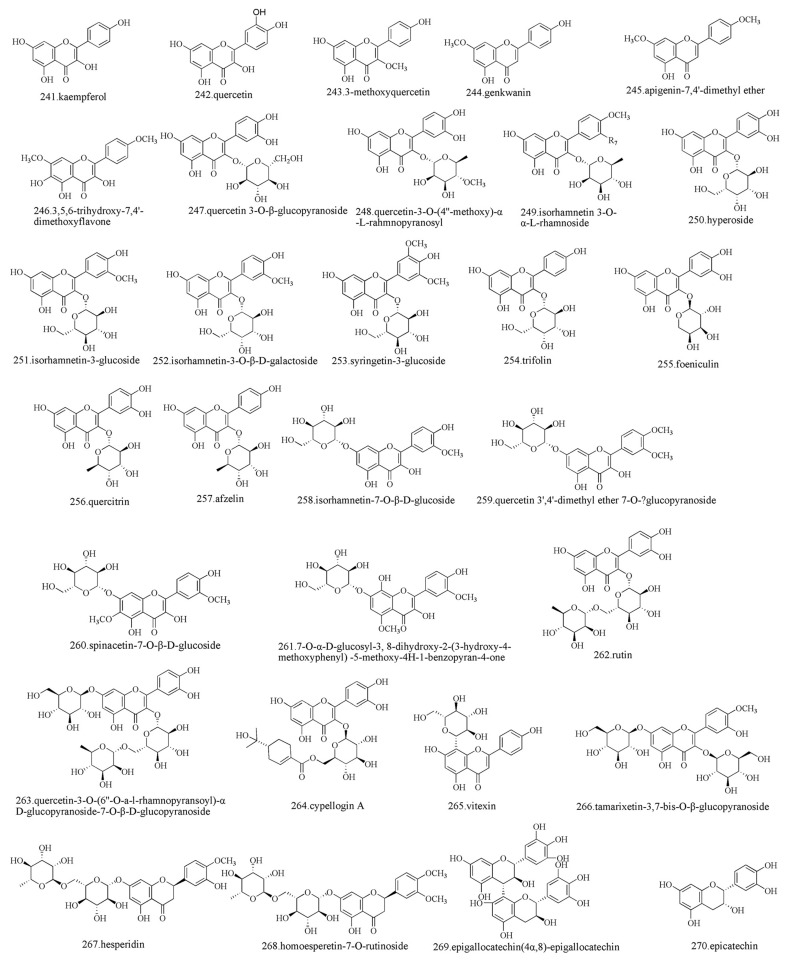
Chemical structures of flavonoids isolated from *Zanthoxylum* [7,9].

Flavonoids are a large group of ubiquitous secondary metabolites with a wide range of biochemical and biological effects [55]. Nine flavonoids have been identified by liquid chromatography–mass spectrometry using methanol extracts from various tissues of *Z. zanthoxyloides* (fruits, leaves, stems, trunk barks, and root barks) [56]. The flavonoids of *Zanthoxylum* are mainly flavanones, flavones, and flavonols, and the most common flavonoid metabolites are hyperoside, quercitrin, kaempferol, afzelin, hesperetin, apigenin, butin, isorhamnetin, and others [57,58,59,60]. Within these flavonoids, hyperoside, isorhamnetin, quercetin, and kaempferol are the main sources of bitterness in the peel of mature green pepper [61]. Compounds such as quercetin and kaempferol found in *Z. piperitum* exhibit antiviral activity against influenza virus A/NWS/33, and these related compounds inhibit the activity of viral neuraminidase in a dose-dependent manner [42]. Flavonoid extracts from *Z. lemairei* demonstrate inhibitory effects against *Escherichia coli* AG100 and *Klebsiella pneumoniae* KP55, with a minimum inhibitory concentration of 64 µg/mL for lermairones B against AG100 [42]. Anthocyanins are a class of flavonoids that confer different colors to *Zanthoxylum* fruits. Recent research comparing the types and contents of anthocyanins in green and red Chinese peppers at different developmental stages shows that the peel contains various anthocyanins, including cyanidin, delphinidin, peonidin, and pelargonidin. Among these anthocyanins, the content of cyanidin is the highest in ripe red Chinese pepper fruits [60]. The main flavonoids isolated from *Zanthoxylum* are shown in Figure 4.

### 3.4. Other Compounds

In addition to alkaloids, flavonoids, and terpenes, *Zanthoxylum* contains a rich array of fatty acids, phenolic acids, and lignans [62]. The primary fatty acids found in *Zanthoxylum* include palmitic acid, oleic acid, linoleic acid, and alpha-linolenic acid [63,64]. Palmitic acid is the most abundant in green pepper, while oleic acid is predominant in red pepper [65]. Furthermore, most *Zanthoxylum* varieties contain chlorogenic acid, quercetin, ferulic acid, quercitrin, and hypericin [66,67]. Notably, ferulic acid and quercitrin are in high abundance in *Zanthoxylum*.

## 4. Research Progress in the Molecular Biology of *Zanthoxylum*

**Figure 5:**
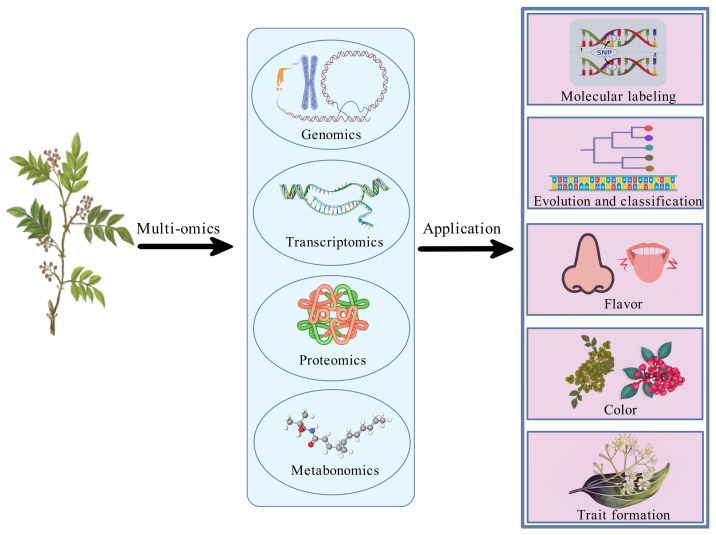
Current molecular biology research strategies and their applications for *Zanthoxylum*.

The genomics and molecular biology research of *Zanthoxylum* has been greatly limited owing to its large genome, high chromosome number, significant heterozygosity, and rich repetitive sequences. This constitutes a highly challenging research field. In recent years, remarkable achievements have been made with the rapid development of genomic sequencing technology and the comprehensive application of genomics, transcriptomics, metabolomics, proteomics, and microbiomics (Figure 5). These achievements helped reveal the genetic diversity, systematic classification, and functional genomics of *Zanthoxylum* species. These landmark studies provide valuable perspectives for the species identification of *Zanthoxylum*, including the analysis of phylogenetic relationships, the development of genome sequencing and assembly, and the exploration of key secondary metabolite biosynthetic pathways.

### 4.1. Application of Molecular Labeling Technology in Zanthoxylum

Molecular marker technology is widely applied in areas such as the genetic diversity, population structure, identification of germplasm resources and breeding of *Zanthoxylum*. The molecular markers currently used in *Zanthoxylum* include amplified fragment length polymorphism (AFLP), random amplified polymorphic DNA (RAPD), sequence-related amplified polymorphism (SRAP), inter-simple sequence repeats (ISSRs), simple sequence repeats (SSRs), chloroplast DNA (cpDNA), and so on. Zheng et al. identified 21 pepper germplasms from Shaanxi, Hebei, Gansu, and Shandong using RAPD technology, and divided them into seven groups through cluster analysis [68]. Li et al. established the ISSR-PCR system in pepper shells for the first time and screened out 18 specific primers, which provided a reference basis to identify *Z*. *dissitum* shell germplasm resources [69]. Dasgupta analyzed the DNA fingerprints of *Z. acanthopodium* and *Z. acanthopodium* using AFLP technology and identified 23 species-specific markers [70]. Genetic diversity analysis of 269 pepper samples collected from five different germplasm regions through SRAP suggests that Shaanxi pepper and Yunnan pepper might have developed different trait characteristics owing to regional differences during the domestication process [71]. Feng et al. identified 182 germplasm samples of *Zanthoxylum* from 18 germplasm origins based on three cpDNA markers of *Zanthoxylum*. Reconstruction of the ancestral distribution area of *Zanthoxylum* shows that its population originates in the Yunnan–Guizhou region. This study provides genetic evidence for the origin and distribution of *Zanthoxylum*, and offers an important reference basis for the phylogenetic and population genetic research of *Zanthoxylum* [72]. Simple sequence repeat analysis using 260 germplasm samples of *Zanthoxylum* from 45 germplasm sources indicates that the genetic diversity of *Zanthoxylum* is higher than that of bamboo-leaf *Zanthoxylum* [73]. The Qinling Mountains are the main geographical obstacle that affects gene exchange between *Zanthoxylum* and bamboo-leaf *Zanthoxylum*. There are significant genetic differentiations between cultivated and wild varieties of *Zanthoxylum*. This study provides a fundamental genetic map to conserve and develop the existing germplasm of *Zanthoxylum*. Subsequently, Li et al. screened and identified 36 polymorphic SSR markers based on whole-genome sequencing of *Zanthoxylum* [74]. The above research provides an important foundation to identify pepper germplasm resources, which can result in genetic improvements and genetic diversity through variety breeding.

### 4.2. Genome Sequencing and Assembly of Zanthoxylum

The large genome of *Zanthoxylum* contains many chromosomes, high heterozygosity, and repetitive sequences, which makes assembly very difficult. Feng et al. used the widely cultivated *Z. bungeanum* to assemble the pepper genome for the first time through whole-genome sequencing [1]. The genome has 68 chromosomes and contains approximately 4.23 Gb of data, with heterozygous rates and duplication rates of about 4.11% and 93.17%, respectively. Further phylogenetic genomic analysis indicated that *Zanthoxylum* and sweet orange were the most closely related, and they diverged approximately 32.9 million years ago. Evolutionary analysis revealed that bamboo-leaf *Zanthoxylum* experienced a whole-genome doubling event approximately 26.6 million years ago, followed by an LTR burst event about 6.4 million years ago. This led to an increase in the genome size of *Zanthoxylum* to 8.5 times that of sweet oranges [75]. Subsequently, a series of chromosome breaks and fusions eventually led to the formation of the current chromosomes. In 2023, a landmark achievement was the assembly of chromosome-level genomes of the widely cultivated tetraploid bambus-leaf pepper and red pepper [15]. The assembly results indicated that the pepper genome had intense genomic rearrangements and two whole-genome replicas, which led to the large genome size (~4.5 Gb), high repetition ratio (>82%), heterozygosity (>6%), and chromosome number (2n = 4 × = 132). The assembly of the pepper genome provides a good reference basis for the formation of its important traits, development of medicinal functions, identification of germplasm resources and molecular directional breeding, and so forth.

### 4.3. Synthetic Biology of Main Flavor Metabolites in Zanthoxylum

Synthetic biology has emerged as an alternative approach to resource acquisition, which can rapidly produce large quantities of bioactive compounds. This method holds broad application prospects in the development and sustainable utilization of medicinal plant resources. Currently, it has successfully produced natural products including artemisinic acid, ginsenosides, and others. The biosynthesis of flavor metabolites in *Zanthoxylum* is also attracting significant attention.

**Figure 6:**
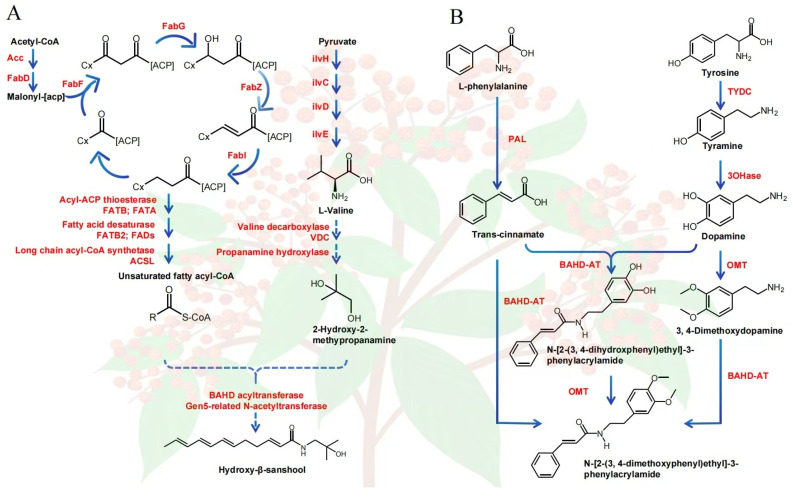
Biosynthesis of two main alkaloids in *Zanthoxylum*. The putative pathways and candidate genes involved in the biosynthesis of hydroxy-β-sanshool [1] (**A**) and N-[2-(3,4-dimethoxyphenyl)ethyl]-3-phenyl-acrylamide (GX-50) [75] (**B**) in *Zanthoxylum*, respectively. The solid lines indicate genes catalyzing major reactions that were characterized. The dotted lines indicate unclear pathways.

Feng et al. deciphered the metabolic pathway of sanshool based on genomic sequencing [1]. Namely, sanshools are directly synthesized from two precursor substrate unsaturated fatty acid fragments and propylamine under the catalysis of a potential acetyltransferase (NAF), and the fatty acid is usually composed of 12c or 14c unsaturated fatty acid acyl coA synthesized by acyl acp thionase and fatty acid desaturase. In contrast, the biosynthesis of propylamine is divided into two steps: valine decarboxylation through branched-chain amino acid (BCAA) decarboxylase, followed by the production of 2-hydroxy-2-methylpropylamine under the action of cytochrome P450 hydroxylase (Figure 6A).

Huang determined changes in the contents of key bitter metabolites in the peel of the relatively bitter Chongqing Jiangjin Jiuye green pepper based on high-performance liquid chromatography (HPLC) analysis [61]. Five flavonoids were identified as the main sources of bitterness. Further analysis of the co-expression network of weighted genes indicates that the expression of 13 structural gene families in *Zanthoxylum* (such as PAL, C4H, 4CL, COMT, F5H, F6H, F3H, F3′5′h, F3′H, DFR, ANS, ANR, and FLS) strongly correlates with the dynamic accumulation of bitter metabolites in the peel of green *Zanthoxylum*. These findings reveal the biosynthetic pathways and biological mechanisms of key bitter metabolites during the growth and development of *Zanthoxylum* peels, and expand the understanding of the sources of bitterness during the growth of *Zanthoxylum* peels, which lays a theoretical foundation for further exploration of the wild-type plant, and may result in the development of methods to remove the bitterness of *Zanthoxylum* peels.

The aromas of green *Zanthoxylum* and red *Zanthoxylum* are mainly due to their menthone and limonene terpenes, respectively, according to gas chromatography–mass spectrometry analysis [76]. In-depth transcriptome analysis shows the key roles of *HDS2*, *MVK2*, and *MVD* genes in the synthesis of terpenoids in *Zanthoxylum*, while *FDPS2* and *FDPS3* genes are crucial for the synthesis of terpenoids in *Zanthoxylum*. The accumulation of these metabolites and the differences in gene expression have led to significant differences in the aroma of different types of *Zanthoxylum*.

### 4.4. Formation Mechanism of Other Qualities in Zanthoxylum

In recent years, studies on the formation rules and biological mechanisms of the main components of *Zanthoxylum* have emerged, as well as its color and asexual reproduction. Wang et al. analyzed two potential biosynthetic pathways of the active alkaloid GX-50 in *Zanthoxylum* based on its chemical molecular structure [75]. Both pathways are related to the production of tyrosine, tyramine, dopamine, L-phenylalanine, and *trans*-cinnamic acid, although the sequence of acyltransferase and methyltransferase reactions differs in the last two steps. After combining HPLC and weighted gene co-expression network analysis, it was inferred that the key gene families involved in the biosynthesis of GX-50 include tyrosine decarboxylase (TYDC), tyrosinase (3OHase), phenylalanine ammonia-lyase (PAL), *O*-methyltransferase (OMT), and BAHD acyltransferase (BAHD-AT) [75] (Figure 6B).

The main fatty acids found in *Zanthoxylum* are palmitic acid, palmitoleic acid, elaidic acid, linoleic acid, and linolenic acid [60]. In the mature stage of *Z. bungeanum*, the content of palmitic acid in its seeds can exceed 40 mg/g. Several studies have identified genes associated with fatty acid synthesis in *Zanthoxylum*, including structural genes involved in the fatty acid synthetic pathway such as fatty acid desaturase (FAD) and the acyltransferase gene family [1,15,63]. However, there is a lack of relevant reports on the detailed regulatory mechanisms of functional genes involved in fatty acid synthesis.

The key anthocyanins during the color change of pepper peel from green to red are geranion-3, 5-*O*-bisglucoside, paeonion-*O*-hexanoside, anthocyanin-*O*-syringic acid, and paeonion-3-*O*-glucoside according to metabolomic analysis of three varieties of *Zanthoxylum* at different color development stages [60]. The accumulation of these anthocyanins is affected by the upregulated expression of *ANS* and *UFGT* genes according to transcriptome sequencing analysis. Members of the MYB transcription factor family are involved in anthocyanin biosynthesis in *Z. bungeanum* [77]. In addition, the biosynthesis of anthocyanins in *Zanthoxylum* involves the COP1–HY5 module, which is a light-responsive element. Ultraviolet rays induce the expression of ZbHY5, which promotes the accumulation of anthocyanins in *Z. bungeanum* leaves [78].

The flowers of *Zanthoxylum* are mainly female, with male flowers and bisexual flowers occasionally seen. Therefore, asexual reproduction is an important biological characteristic. Namely, female plants do not need to be fertilized and directly develop into embryos from the pericardium cells to reproduce offspring. Many male flowers have been produced in *Zanthoxylum planispinum* var. *dintanensis* [79]. The *Zardc07021* gene might play an important role in the asexual reproduction process of pepper plants [75]. However, the detailed molecular basis of asexual reproduction remains to be explored. In the future, the gene functions and formation mechanisms related to asexual reproduction of the genus *Zanthoxylum* will be further clarified with the continuous decline in sequencing costs and the advancement of sequencing and assembly technologies.

## 5. Conclusions and Future Prospects

Metabolites play crucial roles in the growth and development of all plants. Different species have developed specific metabolites through long-term interactions with the environment and domestication. *Zanthoxylum* is a medicinal and edible plant with a unique stress-resistant phenotype and specific metabolites, which could be incorporated as valuable adaptation strategies by other species in increasingly harsh environments. Furthermore, the applications of *Zanthoxylum* can be extended to various industries, such as food, medicine, cosmetics, animal feed, agricultural production, and machinery manufacturing. The recent publication of *Zanthoxylum* genomes (*Z. bungeanum* and *Z. aratum*) and advancements in metabolite identification and separation methods provide a foundation for further exploration of their special metabolites and potential benefits. However, research on *Zanthoxylum* metabolites is in its early stages. Future research should focus on the three main areas discussed below.

(1) To comprehensively identify the metabolites in *Zanthoxylum*, advanced separation and detection technologies are required. Additionally, it is crucial to comprehend how these metabolites are synthesized and regulated and determine where they accumulate in *Zanthoxylum*. Single-cell omics techniques can enhance the analysis of *Zanthoxylum* metabolites. In addition, the synthesis of specific metabolites is closely linked to changes in specific metabolic pathways during evolution. These changes include the regulation of transposons, variations in key gene promoter regions, and tandem duplications of genes. Therefore, it is crucial to utilize multi-omics methods to comprehensively identify key genes in the relevant metabolic pathways and conduct gene function research to study the metabolites of *Zanthoxylum*.

(2) To study the biosynthesis and regulation of characteristic components of *Zanthoxylum*, it is necessary to establish a suitable genetic operating system. Although a series of metabolic enzymes have been identified from *Zanthoxylum*, there are certain limitations on the synthetic pathways of characteristic components owing to the complexity of plant secondary metabolism and the multi-layer nature of regulatory mechanisms. Therefore, in-depth research on the biosynthesis and synthetic biology of the main components is urgently needed. These efforts will lay the foundation for the development of new products and provide innovative solutions to the challenge of sustainable utilization of *Zanthoxylum* resources.

(3) To efficiently utilize *Zanthoxylum* to create capital value, the development of functional products needs to cater to specific market needs. The emergence of plant-specific metabolites is the outcome of a plant’s long-term interaction with the environment, and *Zanthoxylum* contains metabolites with noteworthy biological activities. Further research is needed to explore the interaction of *Zanthoxylum* with various organisms, including humans, and to fully evaluate their medicinal value. This will contribute to a more comprehensive understanding and effective application of these specific metabolites.

## Figures and Tables

**Table 1:** Chinese industrial applications of *Zanthoxylum* products.

Patent Number	Date of Publication	Title	Summary
CN102836260A	7 September 2012	A method for extracting antioxidant active plant polyphenols from *Zanthoxylum* leaves	A method for extracting antioxidant active plant polyphenols from *Zanthoxylum* leaves, which is characterized by crushing and sifting *Zanthoxylum* leaves and adding methanol solution to extract them.
CN106309671B	18 July 2017	Extracts of *Zanthoxylum bungeanum* L. ‘Longnan’, Gansu Province, preparation method and detection method thereof	A plant extract study which relates to an extract of Gansu *Zanthoxylum bungeanum* L. ‘Longnan’ with an anesthetic effect and the preparation method thereof. The extract has a significant effect on the treatment of arthritis and venous injury.
CN103623105A	26 August 2012	Use of *Zanthoxylum* extract in preparation of drugs regulating cholesterol metabolism	A pharmaceutical study which relates to the use of *Zanthoxylum* extract in preparing drugs regulating cholesterol metabolism. It specifically relates to the regulation of cholesterol metabolism using different solvent extracts under the conditions of high cholesterol and inflammation.
CN111568794B	25 August 2020	New application of active ingredient WGX50 in *Zanthoxylum* extract	Relates to a new use of the active ingredient WGX50 in *Zanthoxylum* extract, provides a potential scheme for the preparation of multi-functional cosmetics, opens up a new idea for the preparation of cosmetics, and is conducive to realizing higher commercial value.
CN110279768A	27 September 2019	Use of *Zanthoxylum* in the preparation of drugs for the prevention and/or treatment of neurodegenerative diseases	Demonstrates the use of *Zanthoxylum* in the preparation of drugs for the prevention and/or treatment of neurological diseases. The experimental results show that *Zanthoxylum* can be used in the treatment of neurodegenerative diseases and can be used in pharmaceuticals.
CN101326942A	24 December 2008	Two-step extraction of prickly ash seed skin oil and kernel oil with the same solvent	Discloses a process for extracting *Zanthoxylum* seed oil. After refining, the kernel oil can be used as edible oil, and the skin oil can be used as industrial oil, which can effectively solve the low oil yield and poor quality of kernel oil.
CN112980445B	12 April 2022	Relates to a method for extracting phenolic antioxidants from processing by-products of *Zanthoxylum* oil	Discloses a method for extracting phenolic antioxidants from the by-products of *Zanthoxylum* oil processing. The extraction process can obtain phenolic antioxidants with high purity and high activity, and can also reduce energy consumption, costs and pollution.
CN109453253B	18 June 2021	Relates to a method for extracting flavonoids antioxidants from processing by-products of *Zanthoxylum* oil	Discloses a method for extracting flavonoid antioxidants from the by-products of *Zanthoxylum* oil processing, which can improve the comprehensive utilization rate of raw material waste, reduce pollution, and have positive economic and social benefits.
CN102690208A	26 September 2012	Relates to a method for extracting hydroxy-α-sanshool from *Zanthoxylum* oil	Discloses a method for extracting hydroxy-α-sanshool from *Zanthoxylum* oil which provides a standard product that can provide a quantitative numbing taste, is convenient to operate, and can obtain a high-purity isolate.
CN111718814A	29 September 2020	*Zanthoxylum* craft beer preparation method	Discloses a *Zanthoxylum* craft beer and the preparation method thereof. The prepared beer has a moderate bitter taste, has the numbing taste and fragrance of prickly pepper, and is more acceptable to consumers.
CN113974117A	28 January 2022	*Zanthoxylum* salt microcapsule compound flavor	Outlines a *Zanthoxylum* salt microcapsule compound seasoning and the preparation method thereof, solving the problems of single salt variety, low added value, poor solubility of simple mixed seasoning, etc., which can be applied to the food processing industry.
CN115304508A	8 November 2022	Preparation method of a biomimetic antibacterial and antioxidant nanoparticle based on *Zanthoxylum* fruit extract	Outlines an antibacterial and antioxidant nanoparticle based on *Zanthoxylum* fruit extract and the preparation method and applications thereof. The nanoparticle can remove wound bacteria, promote healing, and be used for clinical treatment.
CN116420504A	14 July 2023	Utility model of a multi-functional green *Zanthoxylum* picking machine	Describes a multi-functional green *Zanthoxylum* picking machine, which can be used for drying, picking, separation of peel/seed, collection, and integrated production.
CN113491647B	20 June 2023	Method for preparing a pain-relieving and anti-inflammatory toothpaste from *Zanthoxylum* herb	Describes an analgesic and anti-inflammatory toothpaste made using *Zanthoxylum* herb and possesses anti-inflammatory, antibacterial, anti-allergic, analgesic, and other effects, and has excellent industrialization prospects.
CN116350547A	30 June 2023	Preparation method of anti-dandruff shampoo containing a nano-emulsion of green *Zanthoxylum* aromatic essential oil	Describes an anti-dandruff shampoo containing a green *Zanthoxylum* aromatic essential oil nano-emulsion, which can enhance the writing effect, delay the generation of drug resistance, and effectively solve the application defects of chemical drugs and natural products.
CN219318990U	7 July 2023	A continuous *Zanthoxylum* heat pump dryer dehumidifying device	Describes a continuous *Zanthoxylum* heat pump dryer humidification system, comprising a humidification channel and a humidification outlet, which can remove water vapor near the central axis of the dryer over time.
CN116252321A	13 June 2023	*Zanthoxylum* picking soft manipulator	Describes a soft manipulator for picking prickly ash, which can wring prickly ash branches to ensure safe picking.

## Data Availability

No new data were created or analyzed in this study.
